# Risk Analysis for Patient Safety in Surgical Departments: Cross-Sectional Design Usefulness

**DOI:** 10.3390/ijerph17072516

**Published:** 2020-04-07

**Authors:** Verónica Aranaz Ostáriz, María Teresa Gea Velázquez de Castro, Francisco López Rodríguez-Arias, José Lorenzo Valencia Martín, Carlos Aibar Remón, Juana Requena Puche, Cristina Díaz-Agero Pérez, Antonio Fernando Compañ Rosique, Jesús María Aranaz Andrés

**Affiliations:** 1 Hospital Universitario Sant Joan d’Alacant. Ctra, N-332, s/n, Sant Joan d´Alacant, 03550 Alicante, Spain; teregea@gmail.com (M.T.G.V.d.C.); af.company@umh.es (A.F.C.R.); 2 Hospital General Universitario de Elche, C/Almazara 11, 03203 Alicante, Spain; franloarias@hotmail.com; 3 Hospital Universitario Ramón y Cajal, IRYCIS. M-607, km 9100, 28034 Madrid, Spain; jose.valencia@salud.madrid.org (J.L.V.M.); cristina.diazagero@salud.madrid.org (C.D.-A.P.); jesusmaria.aranaz@salud.madrid.org (J.M.A.A.); 4 Hospital Clínico Universitario Lozano Blesa, Avda. San Juan Bosco, 15, 50009 Zaragoza, Spain; caibar@unizar.es; 5 Hospital General Universitario de Elda, Ctra, Sax-La Torreta, s/n, Elda, 03600 Alicante, Spain; requena_jua@gva.es; 6 Center for Biomedical Research in the Epidemiology and Public Health Network (CIBERESP), 28029 Madrid, Spain

**Keywords:** adverse events, medical errors, clinical safety, quality of care, patient safety

## Abstract

**(1) Background:** Identifying and measuring adverse events (AE) is a priority for patient safety, which allows us to define and prioritise areas for improvement and evaluate and develop solutions to improve health care quality. The aim of this work was to determine the prevalence of AEs in surgical and medical-surgical departments and to know the health impact of these AEs. **(2) Methods:** A cross-sectional study determining the prevalence of AEs in surgical and medical-surgical departments was conducted and a comparison was made among both clinical areas. A total of 5228 patients were admitted in 58 hospitals in Argentina, Colombia, Costa Rica, Mexico, and Peru, within the Latin American Study of Adverse Events (IBEAS), led by the Spanish Ministry of Health, the Pan American Health Organization, and the WHO Patient Safety programme. **(3) Results:** The global prevalence of AEs was 10.7%. However, the prevalence of AEs in surgical departments was 11.9%, while in medical-surgical departments it was 8.9%. The causes of these AEs were associated with surgical procedures (38.6%) and nosocomial infections (35.4%). About 60.6% of the AEs extended hospital stays by 30.7 days on average and 25.8% led to readmission with an average hospitalisation of 15 days. About 22.4% resulted in death, disability, or surgical reintervention. **(4) Conclusions:** Surgical departments were associated with a higher risk of experiencing AEs.

## 1. Introduction

Since the initial work of Donabedian, we have known that, from a conceptual perspective, quality in health care is an essential component to ensuring a positive clinical outcome [1,2]. However, we now have evidence that “poor medical care” leads to more than five million deaths per year worldwide. It is estimated that the total number of deaths each year from poor quality care is five times higher than the number of global deaths caused by HIV/AIDS (one million) and is more than three times higher than that of deaths caused by diabetes (1.4 million) [3].

Anywhere in the world where health care is provided, it is done so with the goal of providing more benefit than harm, is based on the best available evidence and cost-effective studies, and takes into consideration the needs and circumstances of each patient and their values.

In this regard, in what has been termed ‘right care’ [4], the identification and measurement of adverse events (AEs) is a priority for patient safety that enables the establishment of a hierarchy and a definition of the areas for improvement, as well as the evaluation and development of solutions designed to improve the quality of health care.

To ensure this, it is necessary to perform a continual and selective evaluation that emphasises the identification and analysis of AEs that occur during healthcare provision. This is a priority for patient safety, as it allows us to prioritize and define specific areas for improvement within our organization, and to evaluate the development of solutions designed to improve the quality of our healthcare.

In the past 15 years, a significant number of studies have been published that estimate the frequency of AEs and their characteristics, considering their nature, impact, and the possibilities for prevention [5]. In addition, multiple improvement programmes have been implemented to reduce healthcare-related harm in various settings and health practices. Despite this, AEs have continued to occur and have proven to be a difficult problem to solve [6].

Unwanted healthcare effects represent a considerable source of sickness and mortality [7,8], in addition to having a considerable economic and social impact. The frequency of AEs has been estimated to be between 4% and 17%, of which about 50% are considered to be avoidable [9,10,11,12,13,14,15,16,17,18,19,20]. If we analyse the frequency of AEs in general surgery, this number increases, ranging between 10.3% and 23.2% [9,13,17,21,22,23,24,25,26,27].

The methodology that ideally should be used for these types of epidemiological studies has been widely analysed, with a general consensus that it should be based on the objectives of the study and the need to combine the minimisation of bias and validity to identify AEs with reproducibility of value judgments in their iatrogenic or avoidable nature [21,28].

The 2010 Latin American Study of Adverse Events (IBEAS) [29] was a cross-sectional study with 58 participating hospitals from five Latin American countries (Argentina, Colombia, Costa Rica, Mexico, and Peru), carried out in collaboration with the Health Ministry of Spain, the Pan American Health Organization, and the WHO Patient Safety programme. The cross-sectional design was new and took into account, both, the fewer required resources and the possible subsequent replication of the study. Thus, the IBEAS estimated an AE prevalence of 10.5%, of which approximately 60% were considered avoidable. Of all AEs, 13.4% were related to care, 8.2% to medication, 37.1% to nosocomial infections, 28.5% to a surgical procedure, and 6.1% to diagnoses. In addition, 64.7% of the AEs prolonged patient hospital stays by an average of 16.1 days, 28.8% caused disability, and 5.8% were related to the death of the patient [30].

Despite the inherent limitations of the cross-sectional design, which does not enable the study of the entire course of the hospitalization incidents, this approach is a far more efficient design in terms of time and resources, offering similar results to incidence studies. When repeated, it becomes a systematic monitoring and evaluation instrument for enhancing patient quality and safety improvement programmes [21,31].

The objectives of this study were to determine the prevalence of AEs in the surgical and medical-surgical departments of Latin American hospitals and to draw a distinction between the moment of occurrence of the AE, its immediate causes, and how to prevent said AEs. In addition, this study aimed to determine the impact of AEs in terms of disability, death, or extended hospital stays.

## 2. Materials and Methods

The present study was carried out by means of analysing data extracted from the IBEAS study, a transversal study that used intentional sampling.

The selection of hospitals was opportunistic. The objective of the study was to investigate the existence of problems related to patient safety during care, rather than to obtain national inferences. To be able to compare countries, a sample of at least 2000 patients was taken per country, with a minimum accuracy level of 1.5% for an estimated prevalence rate of 10% and a loss of 5% [30].

The result variables were the prevalence of patients with AEs and the frequency of AEs, since a patient might experience more than one AE during hospitalisation. AE was defined as any event related to healthcare that caused unnecessary harm to the patient [32,33,34]. The sample was obtained by surveying all patients admitted to the hospitals at the time of the study (one week at the end of 2007). AEs had to be present on the day of observation but could have occurred during previous healthcare episodes.

The patients included in this study were those admitted to surgical departments, including the specialties of cardiac surgery, general and digestive surgery, maxillofacial surgery, paediatric surgery, traumatology and orthopaedic surgery, plastic surgery, thoracic surgery, vascular surgery and neurosurgery, and patients admitted to medical-surgical departments, including the specialties of dermatology, gynaecology and obstetrics, ophthalmology, otorhinolaryngology, urology, and others.

All hospitalised patients were screened using an adapted form that had been validated in previous studies [35,36]. The patients with positive screening items were assessed using the MRF2 Questionnaire [37], which evaluated the characteristics of the AE, its involvement in the harm presented by the patient, its impact on patient health and healthcare assistance, and its preventability.

A descriptive analysis was carried out by exploring the distribution of the primary variables through a bivariate analysis, chi-square test, or Fisher’s exact test for categorical variables, and the Student’s *t*-test or the Mann–Whitney U test for numerical variables, alongside the analysis of variance. Lastly, we developed logistic regression models to investigate the factors associated with the occurrence of AEs using independent variables related to hospitalization, patient characteristics, and the characteristics of the AEs.

### Patient and Public Involvement

Neither the patients nor the public were involved in this study and did not actively participate in it. The IBEAS was a cross-sectional study in which researchers used two tools to detect harmful incidents, namely a screening guide and a modular questionnaire, using the medical record review.

First, the screening guide (a questionnaire based on previous studies performed in New York, Utah, and Colorado) was applied to the patients from the IBEAS study by two researchers from each hospital who had been trained for this task. They were studied for 24 h prior to the review, which served as an alert and tracking system for possible incidents. All patients admitted to the hospitals (except those admitted to the emergency department) were studied.

If a patient screened positive for one or more of the 19 criteria in the screening guide, the case was studied using the case history. An in-depth study of the case histories enabled the researchers to conclude whether a patient did in fact present the consequences of a harmful incident (true positive) and, if so, to classify the type of event, its severity, any associated factors, and whether or not the incident could have been avoided. This second confirmatory review was performed by medical doctors with at least five years of clinical experience. A patient could have had more than one AE during the same hospitalization period, and in those cases, the study collected all of them.

Reviewer training was carried out in two stages. First, a workshop was organised to present the study and to train the coordinating teams. Training later continued through a virtual platform designed by the Pan American Health Organization (PAHO)/World Health Organization (WHO), for this purpose. We also organised regularly scheduled conference calls to resolve conceptual or organisational issues. In the second phase, the study coordinators in each country trained the researchers who carried out the field work.

To define an AE, the reviewers used a 6-point scale (1 = no evidence; 6 = certain evidence) where ≥ 4 points were required to consider it positive.

A severe AE was defined as an event associated with death or one that had to be surgically repaired.

Avoidable AEs were defined with a 6-point scale (1 = no evidence; 6 = certain evidence), where ≥ 4 points were required to consider it positive.

To control potential variations in the training of reviewers, a concordance study was carried out. The kappa index was calculated for the inter-observer disagreement and was not weighted against the reference standard of the technical team. A value below 0.4 in the assessment of causality or preventability required training reinforcement.

**Ethics approval and consent to participate:** The study steering committee ensured that the relevant national standards for the protection of human subjects and personal data were respected. The study maintained data anonymity and confidentiality and complied with the ethical principles of the Helsinki Declaration and other related bodies. It was not necessary to obtain individual consent from each patient for the study. The study was approved by the Pan American Health Organization (PAHO) Ethics Review Committee (with number registration PAHOERC 158-THS).

The most important benefit for the participating hospitals was that the experience enabled them to establish a culture of patient safety. The study itself increased awareness of patient safety among the participating health professionals and the research made it possible to pinpoint areas for improvement, thereby putting these hospitals on track for improved safety.

**Availability of data and materials:** The IBEAS study database is available on request from PAHO. Requests must be submited to the principal investigator, Aranaz-Andrés JM (jesusmaria.aranaz@salud.madrid.org).

## 3. Results

**Figure 1:**
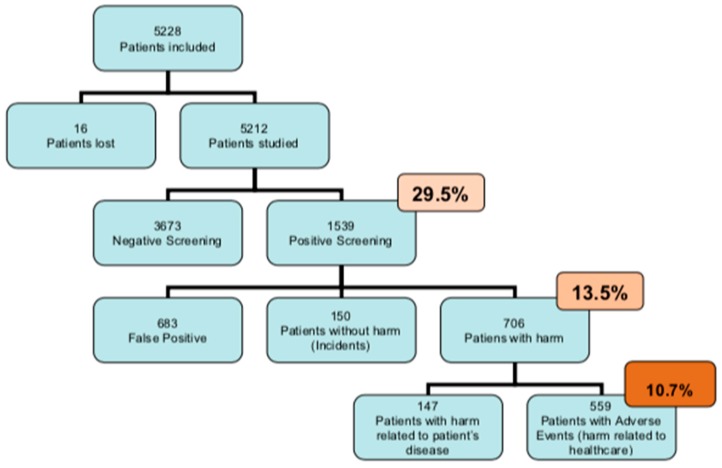
Study patients in the cross-sectional study.

We included 5228 patients who visited the surgical and medical-surgical departments (Figure 1), of which 1539 (29.5%) met at least one selection criterion. Of those patients, 706 (13.6%) experienced some type of injury, although only 559 patients presented with AEs (harm due to healthcare and not due to their pathology). Therefore, the prevalence of AEs was 10.7% (CI 95% 9.9%–11.5%). Data for 16 patients were not included due to loss of medical records. 

The prevalence of AEs by country in surgical and medical-surgical departments, varied from 7.8% (CI 95% 6.2%–9.4%) in country 4 to 16.8% in country 1 (CI 95% 14.3%–19.2%).

On the other hand, 8.8% of the AEs (CI 95% 6.4%–11.1%) occurred prior to hospital admission, while 35.4% (CI 95% 31.5%–39.4%) occurred during a procedure, and 32.4% (CI 95% 28.5%–36.3%) occurred in the hospital room (Table 1). 

When analysing the population characteristics stratified by country of origin, countries 1, 3, and 5 presented a slightly higher median age. The distribution by sex also varied, particularly in country 2 (where the proportion of men was the highest) and in country 3 (where it was the lowest). On the other hand, country 4 presented the highest proportion of elective admissions as well as the shortest average stay (six days). Country 2 had a much higher rate of emergency admissions, but the hospital stay length was similar to that of the other countries. Country 5 presented the longest hospital stays (21 days). Lastly, the proportion of extrinsic risk factors was considerably higher in country 2, while it was far lower in country 3 than in the rest of the countries (Table 2). 

In terms of the comparison between patients with and without AEs, the average age of patients who presented AEs was 45 years (IR 36.5 years) as opposed to the 38 years (IR 34 years) of AE-free patients. AE prevalence was higher in tertiary hospitals than in secondary ones (11.1% vs. 6.7%). Likewise, when incoming patients were admitted as emergency patients, they experienced more AEs (12.1%) than when admission was elective (9.1%). The same was true for the presence of intrinsic risk factors. Patients without intrinsic risk factors had an AE prevalence of 9.0%, as opposed to 20.5% in patients who presented three or more factors.

As for extrinsic risk factors, patients without any factors presented an AE prevalence of 6.4%, which increased dramatically with additional factors (25.5% in patients with three or more factors). Finally, the risk of suffering AEs was also associated with the presence of comorbidity (prevalence was 4.9% in patients without comorbidity, compared to 40.3% for patients who did have additional conditions). All these differences were statistically significant (Table 3). 

The most frequent AEs were those related to surgical procedures (38.6%) and nosocomial infections (35.4%), while other AEs such as medication errors barely represented 4.5%. The most frequent clinical consequences of AEs were surgical wound infections (1.7% CI 95% 1.3%–2.0%), complications following a surgical intervention or a procedure (1.1% CI 95% 0.8%–1.4%), and nosocomial pneumonias (0.60% CI 95% 0.4%–0.9%) (Table 4).

Of the total number of AEs (10.7% CI 95% 9.9%–11.5%), more than half, or 60.2% (CI 95% 55.7%–64.8%) were considered avoidable. On the other hand, 60.6% of the AEs extended patient hospital stays by an average of 30.7 days (SD 15 days). The prevalence of patients whose full admission was due to an AE was 25.8%, with a median of 15 days of hospitalization (IR 10 days). A total of 22.4% (CI 95% 18.9%–25.9%) of the AEs were considered serious (related to death, disability at the time of discharge, or requiring surgical intervention for correction) and 61.2% (CI 95% 57.1%–65.3%) were moderate (Table 5). 

The prevalence of AEs in surgical departments was 11.9% (CI 95% 10.8%–13.1%), higher than that of the medical-surgical departments, which was 8.9% (CI 95% 7.7%–10.2%). The departments with the highest number of treated patients were General and Digestive Surgery and Gynaecology and Obstetrics, which presented an AE prevalence of 11.2% (CI 95% 9.7%–12.6%) and 8.2% (CI 95% 6.9%–9.5%), respectively. On the other hand, Paediatric Surgery was the specialty that presented the highest prevalence of AEs with statistical significance, 23.7% (CI 95% 14.1%–33.2%) (Table 6).

When performing a simple analysis, it was observed that the risk of presenting an AE increased with each day of extended hospital stay. Similarly, being treated in a surgical department, the tertiary complexity of the hospital, emergency hospital admission, and presenting intrinsic and extrinsic risk factors were also factors related to an increased risk of presenting AEs in the simple analysis. Presenting three or more intrinsic risk factors (75% CI 95% 17%–162%) and presenting extrinsic risk factors (65.2% CI 95% 23%–122% with one factor, and up to 382% higher risk with three, CI 95% 222%–620%) showed statistical significance when the analysis was multivariate. Finally, countries 2 and 3 acted as protective factors (0.32 CI 95% 23%–44% and 0.66 CI 95% 46%–95%, lower risk of presenting an AE, respectively) (Table 7). 

## 4. Discussion

This study confirmed the hypothesis that surgical departments constitute an area of particular risk with a significantly higher prevalence of AEs than other clinical settings.

This article is one of many published from the initial IBEAS study in 2007, which are still ongoing. Subsequently, various questions of proven value arose, which required further reflection and analysis, such as the relevance of the prevalence design and the analysis of results in specific areas. We believe that this article is important and exhibits internal and external validity, despite many years having passed since the data collection, due to the expansive check-list, the specific safe surgery programmes in some of the participating countries, the shortage of multicentre and international studies like ours, and the rigour of the method. In addition, the safety of surgical patients was determined by factors associated with the patient’s own vulnerability and factors related to care practice (extrinsic factors), in addition to others related to the system not included in this study.

Our study presents several strengths. First, the large number of patients and participating hospitals and countries. Second, we used previously validated questionnaires from the ENEAS study (National Study of Adverse Events). Third, it is the second-largest study estimating AE prevalence to be conducted in countries with developing economies, together with the study by Wilson et al. [38] Wilson et al. included eight countries from the WHO’s Eastern Mediterranean Regional Office (EMRO) and a total of 15,548 patients, and estimated an AE prevalence of 8.2% (countries ranging between 2.5% and 18.4%), though they did not allow for the calculation of differences between clinical departments as we did. Wilson et al. considered 83% of the AEs to be preventable, while approximately 30% of the AEs were associated with the death of the patient.

Concerning the study limitations, we must highlight those related to the cross-sectional design of the study. This could partly explain some of the findings, since a surgical wound infection is an AE with a longer duration than other types of AEs with a shorter clinical course, meaning that the design would be more likely to identify infections on the day of observation. On the other hand, it would have been very interesting to know the complexity of the surgical procedures to carry out a deeper analysis, but since this was not considered in the initial survey, we did not have the data. However, this opens a new line of work to be taken into account.

The estimated global prevalence of AEs in the IBEAS study was 10.5% [29], a figure that showed little variation upon analysing the grouped surgical and medical-surgical departments from the study, which was at 10.7%. However, when we separated the surgical services from the medical-surgical departments, the prevalence of AEs was 11.9% in the former and 8.9% in the latter, demonstrating the risk difference of the surgical departments with regard to other clinical areas.

These results coincided with the accepted hypothesis that AEs constitute a significant public health problem, as demonstrated in other studies carried out on health systems, in countries with developed economies, and without losing sight of the fact that, as a prevalence study, the risk associated with clinical practice might be underestimated.

In surgical and medical-surgical departments, procedure-related AEs are the primary problem facing patient safety (38.6%); however, surgical site infections are the most prevalent type of AE (15.6% of cases). These results are to be expected, considering that the use of invasive devices, and the presence of surgical wounds, is greater in these clinical settings than in medical departments. On the other hand, the low frequency of medication-related AEs stands out, which other studies have reported as the main patient safety challenge [39]. These differences could be due in part to the design of the study, given that surgical wound infections have a longer average duration than other AEs, thereby, being more easily identifiable in a cross-sectional design.

The rapid development of surgical techniques has resulted in a wider range of indications for invasive treatments, making them feasible in elderly and more fragile patients. A study by Adams et al. showed that the frequency of AEs increases with patient age [40]. Our study found the same phenomenon, however our patients had a median age of 45 years, 20 years younger than those in the study by Adams et al. This was probably due to the fact that our study was carried out in countries with developing economies.

The presence of comorbidity (three or more inherent risk factors) and the use of medical devices (1, 2, 3, or more extrinsic risk factors) are independently associated with a higher risk of presenting an AE, as is the length of the hospital stay, and might potentially be both the origin and the consequence of an AE [41]. However, it should be taken into account that some comorbidities, as well as medical devices, might vary over time within the same patient and the study only exhibited one day of observation.

The proportion of serious AEs in the group of surgical and medical-surgical departments was higher than the proportion reported by the IBEAS study (22.4% vs. 19.8%). We observed the same proportion when focusing on moderate AEs that prolonged hospital stays (61.2% vs. 58.7% in the IBEAS study). Consequently, the proportion of moderately severe AEs was lower (16.4% vs. 21.5%) in the IBEAS study. This reinforced the idea that surgical and medical-surgical departments represent a patient population with increased risk, regardless of patient age or underestimation of the AEs, a finding similar to those of other cross-sectional studies.

Regarding preventability, more than half (60.2%) of AEs were considered avoidable, much like the general results of the IBEAS study (59%). This highlights that preventability does not only depend on the use of instruments that indicate the severity or even the duration of the AE. In any case, this figure is considered to be lower than that of the aforementioned Wilson study [38].

As can be observed, the choice of epidemiological design for the study of AEs is important, and the objectives of the study must be followed, in order to minimise bias and, thus, ensure external validity.

The cross-sectional design was more efficient in terms of time and resources and was easier to carry out, although it did not allow for a complete study of the entire hospitalisation period. This made it more likely to underestimate shorter or milder AEs and overestimate serious AEs or those with a long-term resolution period. Despite this, the cross-sectional design was proven to be more suitable for maintaining a surveillance system, over time. Nevertheless, communication with healthcare staff made it easier to judge the cause of AEs, and the likelihood of preventing them, since the patient was hospitalised at the time of the study.

On the other hand, the fact that the prevalence design proportionally detects more serious AEs is not an inconvenience, as it is precisely these events that we should prioritise when analysing and establishing preventive and control strategies to reduce recurrence [28]. 

It is well-known that surgical settings are a high-risk environment for onset of AEs [42]. In the Harvard Medical Practice Study of New York [11], 48% of the AEs were related to surgical interventions, although medical errors were present in the first place. In the ENEAS study, the specialities with the highest incidence of AEs were Cardiac Surgery (20%), Thoracic Surgery (20%), Vascular Surgery (16.9%), Urology (10.4%), and General Surgery (10.3%).

Other studies have shown a significantly lower incidence of AEs in major ambulatory surgery (MAS) than in General Surgery, with longer hospital stays [43]. This could be explained by the type of patients included in the MAS system—younger patients with fewer comorbidities undergoing less complex procedures. This was worth analysing in-depth, since all surgical departments tended to increase the MAS rate year by year, thus, the severity of patient conditions tended to be similar in both surgery groups.

Interestingly, despite the frequency of AEs being lower in MAS, and considering the increased standardization of the healthcare process, it was more difficult to prevent those AEs that did occur. These details are of vital importance in identifying areas for improvement and guaranteeing patient safety in the different methods of care, as is analysing the clinical practice style. In fact, a significant proportion of AEs (8.8%) occur prior to hospitalisation, although most occur during a procedure (35.4%), or during hospital admission (32.4%). The immediate causes of AEs are directly related to surgical procedures (38.6%) and nosocomial infections (35.4%), while less frequent causes include diagnostic errors (8.2%), medical care-related AEs (6.4%), or medication-related AEs (4.5%). Likewise, and as has been shown in the various studies performed, more than half of AEs (60.2%) are avoidable, and therefore, preventable [18].

Lastly, in assessing the impact caused by AEs, we can compare the fact that 60.6% cause hospital stay extensions with an average of 30.7 days, and 25.8% cause readmission with an average hospitalisation period of 15 days, which offers an idea of the economic impact associated with AEs. If the economic impact is important, so is the burden of illness associated with AEs, given that one in five AEs results in death, disability at the time of discharge, or surgical reintervention.

The IBEAS study served to put the problem of the magnitude of AEs on the agenda of the professionals, managers, and policymakers of the participating countries. The present study reveals the need to continue with already established specific improvement actions for the safety of surgical patients and to propose new initiatives aimed at the effective management of surgical risks and the ‘right care’ in this area.

The most effective strategies for reducing AEs associated with surgical practice are the implementation of surgery safety checklists (“Surgical Checklist”), compliance with clinical protocols [44], and learning through simulation techniques [45]. The WHO safe surgery checklist has reduced morbidity and mortality after surgery [46,47]. In Sweden, this checklist has been expanded by the SURPASS (SURgical PAtient Safety System) protocol [48], which covers everything from surgical treatment decision-making to the post-operative patient revision in consultation. This protocol has managed to reduce the incidence of post-surgical complications and mortality by about 50% [49]. Other strategies of proven utility are the introduction of an electronic medical history to prevent errors in surgical programming [50,51,52]; providing the patient with an informed consent form for intervention in preoperative consultation [53]; marking of the surgical site—if possible with the collaboration of the patients themselves [54]; viewing CT scans, X-rays, or other images [55] during the intervention, to prevent errors in the anatomical area to be treated; correct positioning of the patient on the operating table to prevent secondary vascular or neurological injuries [56]; patient education when being discharged with ostomies, catheters, drains, or other medical devices, to prevent readmissions or new consultations [57], among other actions.

## 5. Conclusions

We can conclude that surgery increases the risk of experiencing AEs, with these being more frequent in surgical departments than in medical-surgical departments. Therefore, knowing this higher prevalence, AEs are related to procedures and surgical wound infections. The relationship between AEs and the modifiable and known risk factors make it both possible and necessary to broaden intervention programmes for the improvement of public health. Such programmes have already demonstrated their effectiveness in these countries, as well as in surgical environments, which have been singled out and flagged for their increased risk in terms of AEs. Lastly, current cross-sectional studies are relatively simple and economical. They should be implemented systematically, following the implementation of these improvement programmes, in order to assess their effectiveness and to identify new objectives and areas for improvement. 

## Figures and Tables

**Table 1:** Moment when the adverse events (AEs) occurred.

		n	%	95% CI
Adverse Events	BEFORE ADMISSION	49	8.8	(6.4–11.1%)
	IN THE ADMISSION TO THE BUILDING	35	6.3	(4.3–8.3%)
	DURING A PROCEDURE	198	35.4	(31.5–39.4%)
	POST-PROCEDURE	61	10.9	(8.3–13.5%)
	AT THE END OF ADMISSION AND GETTING MEDICAL CLEARANCE	24	4.3	(2.6–6.0%)
	IN ROOM	181	32.4	(28.5–36.3%)
	UNKNOWN	11	2.0	(0.8–3.1%)

**Table 2:** Characteristics of study population by country *.

		Country 1		Country 2		Country 3		Country 4		Country 5	
		n	%	n	%	n	%	n	%	n	%
Sex	Female	464	14.6	905	28.4	473	14.9	717	22.5	623	19.6
	Male	436	21.5	570	28.1	264	13.0	356	17.6	402	19.8
Age	Mean (SD)	45.8	21.8	37.8	18.8	43.0	20.2	40.3	21.5	48.9	22.3
	Median (IR)	44	38	33	27	38.5	34	36	32	48	38
Hospital complexity	Tertiary	900	19.0	1276	27.0	737	15.6	794	16.8	1027	21.7
	Secondary (with surgery and ICU wards)	0	0.0	199	41.6	0	0.0	279	58.4	0	0.0
Admission type	Unplanned admission	554	16.5	1032	30.7	521	15.5	542	16.1	714	21.2
	Planned admission	142	10.8	321	24.5	166	12.7	382	29.1	301	22.9
Intrinsic risk factors	No	509	17.2	830	28.1	447	15.1	558	18.9	607	20.6
(coma, renal failure, diabetes, neoplasia, COPD, immunodeficiency, neutropenia, cirrhosis, drug addiction, obesity, malnutrition, pressure ulcer, malformations, heart failure, coronary heart disease, hypertension)	Yes	391	17.3	645	28.5	290	12.8	515	22.8	420	18.6
Number of intrinsic risk factors	0	509	17.2	830	28.1	447	15.1	558	18.9	607	20.6
	1	220	17.3	343	27.0	196	15.4	269	21.1	244	19.2
	2	98	15.2	202	31.4	59	9.2	154	24.0	130	20.2
	3 or more	73	21.1	100	28.9	35	10.1	92	26.6	46	13.3
	Extrinsic risk factors	No	137	9.4	275	18.8	385	26.3	230	15.7	435	29.8
(open urinary catheter, closed urinary catheter, peripheral venous catheter, central catheter by peripheral insertion, central venous catheter, parenteral nutrition, enteral nutrition, nasogastric tube, percutaneous esophagogastric catheter (PEG), tracheostomy, mechanical ventilation, immunosuppressive therapy).	Yes	763	20.3	1200	32.0	352	9.4	843	22.5	592	15.8
Number of extrinsic risk factors	0	137	9.4	275	18.8	385	26.3	230	15.7	435	29.8
	1	436	18.5	750	31.8	291	12.3	461	19.6	419	17.8
	2	176	19.3	322	35.3	46	5.0	236	25.9	131	14.4
	3 or more	151	31.3	128	26.6	15	3.1	146	30.3	42	8.7
Patient comorbidity	No	676	15.5	1300	29.9	620	14.3	867	19.9	885	20.4
	Yes	224	25.9	175	20.3	117	13.5	206	23.8	142	16.4
Number of patient comorbidity	0	676	15.5	1300	29.9	620	14.3	867	19.9	885	20.4
	1	6	33.3	7	38.9	2	11.1	2	11.1	1	5.6
	2	105	25.9	84	20.7	58	14.3	83	20.5	75	18.5
	3 or more	113	25.6	84	19.0	57	12.9	121	27.4	66	15.0
Length of stay until the day of study	Mean (SD)	10.5	21.0	10.8	19.3	11.1	27.9	6.3	16.5	21.2	82.9
	Median (IR)	5	8	4	9	4	10	2	4	7	17

* Missing values are not included in this table.

**Table 3:** Characteristics of the study population by patients with (n = 433) and without AEs (n = 3465) *.

		Patients without AE		Patients with AE		Chi-Square
		n	%	n	%	*p*-Value
Sex	Female	2860	89.9	322	10.1	0.075
	Male	1791	88.3	237	11.7	
Age **	Mean (SD)	42.2	21.1	46.5	21.5	<0.001
	Median (IR)	38	34	45	36.5	
Hospital complexity	Tertiary	4 207	88.9	527	11.1	0.003
	Secondary (with surgery and ICU wards)	446	93.3	32	6.7	
Admission type	Unplanned admission	2957	87.9	406	12.1	0.003
	Planned admission	1193	90.9	119	9.1	
Intrinsic risk factors	No	2687	91.1	264	9.0	<0.001
	Yes	1966	87.0	295	13.1	
Number of intrinsic risk factors ***	0	2687	91.1	264	9.0	<0.001
	1	1120	88.1	152	12.0	
	2	571	88.8	72	11.2	
	3 or more	275	79.5	71	20.5	
Extrinsic risk factors	No	1368	93.6	94	6.4	<0.001
	Yes	3285	87.6	465	12.4	
Number of extrinsic risk factors ***	0	1368	93.6	94	6.4	<0.001
	1	2124	90.1	233	9.9	
	2	802	88.0	109	12.0	
	3 or more	359	74.5	123	25.5	
Patient comorbidity	No	4137	95.2	211	4.9	<0.001
	Yes	516	59.7	348	40.3	
Number of patient comorbidity	0	4137	95.2	211	4.9	<0.001
	1	11	61.1	7	38.9	
	2	245	60.5	160	39.5	
	3 or more	260	59.0	181	41.0	
Length of stay until the day of study **	Mean (SD)	11.0	25.5	29.8	112.5	<0.001
	Median (IR)	4	9	10.5	23	

* Missing values are not included in this table. ** Kruskall–Wallis test. *** Test for trend.

**Table 4:** Adverse event types and proportion of total AEs.

Type of AE	Adverse Event	n	Prevalence (%)	Prevalence (95% CI)	% FREQ
**Related to procedures**	Bleeding or hematoma related to surgery or procedure	27	0.52	(0.32–0.71%)	4.8
	Organ injury during a procedure	29	0.56	(0.35–0.76%)	5.2
	Other complications after surgery or procedure	59	1.13	(0.84–1.42%)	10.6
	Ineffective or incomplete surgical intervention	22	0.42	(0.25–0.60%)	3.9
	Uterine tear	7	0.13	(0.03–0.23%)	1.3
	Pneumothorax	2	0.04	(0.00–0.09%)	0.4
	Suspension of the surgery	8	0.15	(0.05–0.26%)	1.4
	Eventration o evisceration	5	0.10	(0.01–0.18%)	0.9
	Dehiscence of the suture	12	0.23	(0.10–0.36%)	2.1
	Local complications from radiotherapy	1	0.02	(0.00–0.06%)	0.2
	Seroma	3	0.06	(0.00–0.12%)	0.5
	Adhesions and functional alterations after surgery	3	0.06	(0.00–0.12%)	0.5
	Phlebitis	8	0.15	(0.05–0.26%)	1.4
	Others related to a procedure	30	0.58	(0.37–0.78%)	5.4
	**TOTAL**	**216**	**4.14**	**(3.60–4.69%)**	**38.6**
**Healthcare associated infections**	Surgical site infection	87	1.67	(1.32–2.02%)	15.6
	Urinary tract infection	16	0.31	(0.16–0.46%)	2.9
	Another type of nosocomial infection or nosocomial infection without specifying	27	0.52	(0.32–0.71%)	4.8
	Sepsis and Septic Shock	9	0.17	(0.06–0.29%)	1.6
	Nosocomial pneumonia	33	0.63	(0.42–0.85%)	5.9
	Bacteremia associated with device	9	0.17	(0.06–0.29%)	1.6
	Others related to nosocomial infection	17	0.33	(0.17–0.48%)	3.0
	**TOTAL**	**198**	**3.80**	**(3.28–4.32%)**	**35.4**
**Diagnostic issues**	Diagnosis Delay	20	0.38	(0.22–0.55%)	3.6
	Diagnostic Error	23	0.44	(0.26–0.62%)	4.1
	Others related to diagnosis	3	0.06	(0.00–0.12%)	0.5
	**TOTAL**	**46**	**0.88**	**(0.63–1.14%)**	**8.2**
**Care provided**	Pressure ulcer	12	0.23	(0.10–0.36%)	2.1
	Burns, erosions, and contusions (including consequential fractures)	1	0.02	(0.00–0.06%)	0.2
	Lung edema and respiratory failure	3	0.06	(0.00–0.12%)	0.5
	Others related to care	19	0.36	(0.20–0.53%)	3.4
	Phlebitis	1	0.02	(0.00–0.06%)	0.2
	**TOTAL**	**36**	**0.69**	**(0.47–0.92%)**	**6.4**
**Medication**	Nausea, vomiting, or diarrhea secondary to medication	1	0.02	(0.00–0.06%)	0.2
	Itching rash or reactive dermal lesions to drugs or dressings	1	0.02	(0.00–0.06%)	0.2
	Worsening renal function	2	0.04	(0.00–0.09%)	0.4
	Delay in treatment	6	0.12	(0.02–0.21%)	1.1
	Neutropenia	1	0.02	(0.00–0.06%)	0.2
	Drug hypotension	1	0.02	(0.00–0.06%)	0.2
	Opportunistic infection by immunosuppressive treatment	2	0.04	(0.00–0.09%)	0.4
	Ineffective medical treatment	6	0.12	(0.02–0.21%)	1.1
	Others related to the Medication	5	0.10	(0.01–0.18%)	0.9
	**TOTAL**	**25**	**0.48**	**(0.29–0.67%)**	**4.5**
**Others**	**Others AEs**	**25**	**0.48**	**(0.29–0.67%)**	**4.5**

**Table 5:** Impact of AEs (n = 559) *.

		n	%	95% CI
Prolonged hospital stay	No	70	13.6	(10.6–16.5%)
	Yes	312	60.6	(56.4–64.8%)
Extra days same hospitalization	Mean (SD)	14.9	30.7	
	Median (IR)	8	12	
Causing admission	Yes	133	25.8	(22.0–29.6%)
Extra days new hospitalization	Mean (SD)	17.7	25.6	
	Median (IR)	10	15	
Severity	Mild	90	16.4	(13.3–19.5%)
	Moderate	336	61.2	(57.1–65.3%)
	Severe	123	22.4	(18.9–25.9%)
Preventable	No	177	39.8	(35.2–44.3%)
	Yes	268	60.2%	(55.7–64.8%)

* Missing values are not included in this table.

**Table 6:** Prevalence of AEs by department (n = 5212 patients).

	Patients without AE			Patients with AE		
	n	%	95% CI	n	%	95% CI
Cardiac Surgery	6	50.0	(21.7–78.3%)	6	50.0	(21.7–78.3%)
General and Digestive Surgery	1620	88.8	(87.4–90.3%)	204	11.2	(9.7–12.6%)
Maxillofacial Surgery	31	93.9	(85.8–102.1%)	2	6.1	(-2.1–14.2%)
Traumatology and Orthopedic Surgery	662	89.2	(87.0–91.5%)	80	10.8	(8.6–13.0%)
Pediatric Surgery	58	76.3	(66.8–85.9%)	18	23.7	(14.1–33.2%)
Plastic Surgery	52	92.9	(86.1–99.6%)	4	7.1	(0.4–13.9%)
Thoracic Surgery	65	86.7	(79.0–94.4%)	10	13.3	(5.6–21.0%)
Vascular Surgery	31	79.5	(66.8–92.2%)	8	20.5	(7.8–33.2%)
Dermatology	18	100.0	(100.0–100.0%)	0	0.0	(0.0–0.0%)
Gynecology and Obstetrics	1576	91.8	(90.5–93.1%)	141	8.2	(6.9–9.5%)
Neurosurgery	223	84.8	(80.5–89.3%)	40	15.2	(10.9–19.6%)
Ophthalmology	31	93.9	(85.8–102.1%)	2	6.1	(-2.1–14.2%)
Otolaryngology	25	86.2	(73.7–98.8%)	4	13.8	(1.2–26.3%)
Urology	209	89.3	(85.4–93.3%)	25	10.7	(6.7–14.6%)
Others	46	75.4	(64.6–86.2%)	15	24.6	(13.8–35.4%)

**Table 7:** Correlates of adverse events in univariate and multiple logistic regression.

	Univariate			Multivariate (n = 3242)		
Variables	OR	95% CI for OR	*p*-Value	OR	95% CI for OR	*p*-Value
**Length of stay until the day of study**	1.01	(1.01–1.02)	≤0.001	1.01	(1.00–1.01)	≤0.001
**Department (Surgery)**	1.38	(1.15–1.66)	0.001	1.03	(0.81–1.32)	0.801
**Sex (Men)**	1.18	(0.98–1.40)	0.075	0.87	(0.69–1.10)	0.236
**Hospital complexity (Tertiary)**	1.75	(1.21–2.53)	0.003	0.67	(0.30–1.48)	0.320
**Type of admission (Urgent)**	1.38	(1.11–1.71)	0.004	1.17	(0.90–1.53)	0.234
**Number of intrinsic risk factor (none)**	1.00			1.00		
1	1.38	(1.12–1.71)	0.003	1.29	(1.00–1.68)	0.052
2	1.28	(0.97–1.69)	0.076	0.85	(0.59–1.23)	0.391
≥3	2.63	(1.97–3.51)	≤0.001	1.75	(1.17–2.62)	0.006
**Number of extrinsic risk factor (none)**	1.00			1.00		
1	1.60	(1.24–2.05)	≤0.001	1.65	(1.23–2.22)	0.001
2	1.98	(1.48–2.64)	≤0.001	2.15	(1.50–3.10)	≤0.001
≥3	4.99	(3.72–6.68)	≤0.001	4.82	(3.22–7.20)	≤0.001
**Country (Country 1)**	1.00			1.00		
Country 2	0.37	(0.28–0.48)	≤0.001	0.32	(0.23–0.44)	≤0.001
Country 3	0.49	(0.36–0.66)	≤0.001	0.66	(0.46–0.95)	0.025
Country 4	0.42	(0.32–0.56)	≤0.001	0.63	(0.23–1.74)	0.370
Country 5	0.90	(0.70–1.14)	0.373	1.00	(0.74–1.34)	0.979
